# Assessment of metacognitive beliefs in an at risk mental state for psychosis: A validation study of the Metacognitions Questionnaire‐30

**DOI:** 10.1002/cpp.2301

**Published:** 2018-06-07

**Authors:** Measha Bright, Sophie Parker, Paul French, Anthony P. Morrison, Sarah Tully, Suzanne L.K. Stewart, Adrian Wells

**Affiliations:** ^1^ School of Health Sciences, Division of Psychology & Mental Health The University of Manchester Manchester UK; ^2^ Greater Manchester Mental Health NHS Foundation Trust Manchester UK; ^3^ Department of Psychology University of Chester Chester UK

**Keywords:** at risk for psychosis, ARMS, MCQ‐30, metacognitive beliefs, psychosis, validity

## Abstract

**Aim:**

The Metacognitions Questionnaire‐30 (MCQ‐30) has been used to assess metacognitive beliefs in a range of mental health problems. The aim of this study is to assess the validity of the MCQ‐30 in people at risk for psychosis.

**Methods:**

One hundred eighty‐five participants meeting criteria for an at risk mental state completed the MCQ‐30 as part of their involvement in a randomized controlled trial. Confirmatory and exploratory factor analyses were conducted to assess factor structure and construct validity.

**Results:**

Confirmatory factor analyses confirmed the original five‐factor structure of the MCQ‐30. Examination of principal component analysis and parallel analysis outputs also suggested a five‐factor structure. Correlation analyses including measures of depression, social anxiety, and beliefs about paranoia showed evidence of convergent validity. Discriminant validity was supported using the normalizing subscale of the beliefs about paranoia tool.

**Conclusions:**

The MCQ‐30 demonstrated good fit using the original five‐factor model, acceptable to very good internal consistency of items was evident and clinical usefulness in those at risk for psychosis was demonstrated.

**Key Practitioner Message**

- Multicomponent five‐factor structure of MCQ‐30 was confirmed in an ARMS sample.
- Principal components analysis and parallel analysis suggested retaining a five‐factor solution.
- Internal consistency of the MCQ‐30 in ARMS was very good overall.
- The MCQ‐30 correlated meaningfully with related concepts.

## 1 INTRODUCTION

Metacognition is loosely defined as cognition about cognition or thinking about thinking (Flavell, 1979). For example, in the field of memory research, a distinction can be made between cognition (e.g., what can be retained) and the processes of using rehearsal strategies to enhance memory, which requires metacognitive knowledge of what can improve memory. Nelson and Narens (1990) stipulated metacognition involved two levels: the object level where cognition occurs and a metalevel where metacognitive processes occur (Nelson & Narens, 1990) with monitoring and control operations representing the flow of information between these levels.

The importance of a distinction between cognition and metacognitions has been developed in the self‐regulatory executive function (S‐REF) model (Wells & Davies, 1994) of psychopathology. In this model, a syndrome of perseverative thinking is thought to cause most types of psychological disorder. This cognitive attention syndrome (CAS) is a process of worry, rumination, fixating attention on threat, and unhelpful coping behaviours (e.g., avoidance, trying to control thoughts, substance use) and leads to the maintenance of distressing emotions or cognitions. The CAS is linked to underlying metacognitive knowledge (beliefs) that compromise flexible control of the syndrome (Wells, 2009).

In the S‐REF model, metacognitive beliefs principally relate to a subset of positive and negative beliefs people hold about their thoughts. In order to test the model, Wells and colleagues developed a range of measures of metacognitions and the CAS. The gold standard measure of metacognitive beliefs is the Metacognitions Questionnaire (MCQ; Wells & Cartwright‐Hatton, 2004). In a recent meta‐analysis of MCQ studies, metacognitive beliefs were confirmed as transdiagnostic factors across psychopathologies (Sun, Zhu, & So, 2017). Robust and reliable positive associations have been demonstrated between MCQ metacognition domains and symptoms of anxiety (Wells, 2005) and mood disorders (Papageorgiou & Wells, 2003), and more recently, this has been extended to psychotic symptoms (Morrison, French, & Wells, 2007; Sellers, Gawęda, Wells, & Morrison, 2016). However, although the psychometric properties and the construct validity of the MCQ is reasonably well established in nonpatients and those with emotional disorder, relatively little is known about its properties in patients with psychosis or at risk of psychosis, and further advances in this area depend on the interpretability of the measure in psychosis groups. Although the subscales of the MCQ relate to positive and negative beliefs about worry and this measure should be relevant to those experiencing psychosis or who are at risk for psychosis in keeping with the S‐REF model, the latent structure should be confirmed. Paranoia, for example, can be conceptualized as a type of worry with those experiencing such thoughts having positive beliefs about the benefits of worrying (e.g., to protect oneself from harm). Engaging in such thoughts could lead to unhelpful ways of coping (e.g., avoidance of social situations to protect the self). An important step in testing the metacognitive model and treatment applied to psychosis is to determine the properties of the MCQ as an appropriate tool that can be interpreted in the usual way.

The MCQ (Cartwright‐Hatton & Wells, 1997) was originally constructed as a 65‐item measure of metacognitive beliefs and monitoring. The MCQ was developed using data obtained from individuals with generalized anxiety disorder, obsessive compulsive disorder, hypochondriasis, and panic disorder (Cartwright‐Hatton & Wells, 1997). The MCQ has five subscales: positive beliefs about worry (e.g., “Worrying helps me avoid problems in the future.”); negative beliefs about uncontrollability and danger of worry (e.g., “My worrying is dangerous for me.”); negative beliefs about thoughts in general including items relating to superstition, punishment, and responsibility (e.g., “If a bad thing happens which I have not worried about, I feel responsible.”); cognitive self‐consciousness (e.g., “I am constantly aware of my thinking.”); and cognitive confidence (e.g., “I have a poor memory.”).

The original MCQ had limited use due to its length and some items were found to be unclear to participants (Wells & Cartwright‐Hatton, 2004). The Metacognitions Questionnaire‐30 (MCQ‐30; Wells & Cartwright‐Hatton, 2004), a shortened 30‐item version of the MCQ, was developed as a result. The number of items was reduced by removing any items that participants questioned on the original MCQ and by keeping the highest loading items for each subscale (Wells & Cartwright‐Hatton, 2004). The MCQ‐30 was found to have subscales consistent with the original MCQ (Wells & Cartwright‐Hatton, 2004). Internal consistency for the MCQ‐30 subscales was better overall than the original MCQ. MCQ and MCQ‐30 Cronbach alphas (respectively) were as follows: cognitive confidence = 0.84:0.93, positive beliefs = 0.87:0.92, cognitive self‐consciousness = 0.72:0.92, negative beliefs about uncontrollability and danger = 0.89:0.91, and negative beliefs about thoughts in general or negative beliefs about the need to control thoughts = 0.74:0.72. Both the MCQ and MCQ‐30 have sufficient internal consistency as scores are above 0.70 and less than 0.95 (Terwee et al., 2007). The improved internal consistency combined with the more efficient length makes the MCQ‐30 the measure of choice for metacognitive beliefs.

At risk mental state (ARMS) refers to people who are at risk for psychosis. Enhanced interest in the presence of metacognitive beliefs in the early stages of psychosis has led to increased use of the MCQ‐30 in research (Cotter, Yung, Carney, & Drake, 2017; Morrison et al., 2014; Palmier‐Claus, Dunn, Taylor, Morrison, & Lewis, 2013; Welsh, Cartwright‐Hatton, Wells, Snow, & Tiffin, 2014). A systematic review and meta‐analysis of metacognitive beliefs in those with an ARMS (Cotter et al., 2017) found those at risk of psychosis have significantly higher scores compared to healthy controls on all metacognitive belief domains. No significant differences were found between ARMS and those experiencing psychosis on any of the metacognitive belief subscales. Research in this area is helpful in building a picture of the presence of metacognitive beliefs in ARMS and established psychosis, which could help clinicians and researchers work out improved targets for intervention. It is important, therefore, that the tool used to measure metacognitive beliefs is appropriate for the population in which it is being used.

Although the MCQ‐30 has been validated in non‐clinical (Spada, Mohiyeddini, & Wells, 2008; Wells & Cartwright‐Hatton, 2004), obsessive–compulsive disorder (Grøtte et al., 2016), and physical health (Cook, Salmon, Dunn, & Fisher, 2014; Fisher, Cook, & Noble, 2016) populations, no studies have validated the MCQ‐30 in those at risk for psychosis. With the increased use of this measure with those at risk for psychosis, it is important to explore the validity the MCQ‐30 in this population. This study aims to do so by examining the construct validity via the factor structure of the MCQ‐30 and its internal consistency in those with an ARMS.

To examine convergent and discriminant validity, correlations with related measures are useful (DeVon et al., 2007). Although the MCQ measures beliefs about repetitive negative thinking in the form of worry, other forms of similar thinking have been identified in psychosis, such as paranoid ideation, that would be conceptualized as a type of worry in the metacognitive (S‐REF) model. Therefore, negative and positive beliefs about worry (MCQ‐30) should correlate with negative and positive beliefs about paranoia in the current sample, thus providing a means of evaluating convergent validity. It is predicted, therefore, that significant positive correlations will exist between negative beliefs subscales of the MCQ‐30 and the negative beliefs about paranoia subscale of the Beliefs about Paranoia Scale (BAPS; Gumley, Gillan, Morrison, & Schwannauer, 2011). Further, it was hypothesized that significant positive correlations would exist between the BAPS survival subscale (includes items related to positive beliefs about paranoia) and the positive beliefs about worry subscale of the MCQ‐30. It is expected that the BAPS normalizing subscale will have no significant relationships with any of the MCQ‐30 subscales. Due to past research on the relationship between metacognitive beliefs and depression (Brett, Johns, Peters, & McGuire, 2009; McEvoy, Mahoney, Perini, & Kingsep, 2009; Wells, 2009) and social anxiety (Gkika, Wittkowski, & Wells, 2017; Wells, 2009), it is predicted that significant positive relationships will exist between the MCQ‐30 and these areas of emotion measured using the Social Interaction Anxiety Scale (SIAS) and Beck Depression Inventory‐7 (BDI‐7).

## 2 METHODS

### 2.1 Participants

Data from 185 participants meeting criteria for an ARMS were used to conduct this study. Participants were taking part in either the Early Detection and Intervention Evaluation (EDIE; Morrison et al., 2004) or the Early Detection and Intervention Evaluation 2 (EDIE‐2) trial (Morrison et al., 2012). Both were randomized controlled trials testing the efficacy of cognitive therapy in preventing transition to psychosis. Participants who took part in the EDIE‐2 trial were a completely separate sample to those who took part in EDIE (i.e., checks at entry to the EDIE‐2 trial ensured that there was no chance any EDIE participants also took part in EDIE‐2). Participants were recruited from primary (e.g., psychological services and general practitioners) and secondary (e.g., early intervention for psychosis or community mental health teams) care National Health Service (NHS: publicly funded healthcare providers in the UK) services, as well as other non‐NHS services such as university counselling services or voluntary agencies. Research assistants trained in administering all measures collected data for both studies. Thirty‐two participants were drawn from the EDIE trial and 153 from EDIE‐2. The male to female ratio was 112:73.

Ethical approval for EDIE and EDIE‐2 was sought from UK‐based ethical committees. Please refer to the full texts for EDIE and EDIE‐2 for more information (Morrison et al., 2004; Morrison et al., 2012). All participants voluntarily consented to take part in the studies and for anonymous data to be collected and used in publications. Research procedures in both trials were in accordance with the Declaration of Helsinki and Good Clinical Practice guidelines.

### 2.2 Measures and procedures

The current study utilized data from two separate samples (EDIE and EDIE‐2). In EDIE‐2, the MCQ‐30 (Wells & Cartwright‐Hatton, 2004) was used, whereas in EDIE, the 65‐item MCQ was used (Cartwright‐Hatton & Wells, 1997). For the purposes of this study, only the 30 items of the MCQ‐30 were extracted for analysis. It is possible EDIE participants could have been influenced by the additional items in the MCQ when completing the MCQ‐30 items leading to a bias in responses. Separate means and standard deviations (*SD*s) were, therefore, calculated for all five subscales of the MCQ‐30 as well as the total measure (Table 1). Means and *SD*s were found to be similar in both the EDIE and EDIE‐2 samples. A one‐way multivariate analysis of variance (MANOVA) found no significant differences between the means on any of the subscales or total measure. It appeared, therefore, that completing the 30 items of the MCQ‐30 within the larger MCQ item set did not bias EDIE participant responses to these items or the content and face validity of the MCQ‐30.

**Table 1:** Comparison of MCQ‐30 means and SDs for EDIE and EDIE‐2 data

MCQ‐30 Subscale	EDIE (*n* = 32)	EDIE‐2 (*n* = 153)
Cognitive confidence	11.78 (5.17)	12.76 (4.91)
Positive beliefs about worry	10.25 (3.12)	10.46 (4.18)
Cognitive self‐consciousness	14.41 (4.29)	15.96 (4.33)
Negative beliefs about uncontrollability and danger	14.34 (4.05)	15.17 (4.98)
Negative beliefs about the need to control thoughts	11.72 (3.42)	13.24 (4.35)
Total score	62.50 (12.95)	67.59 (16.17)

*Note*. EDIE: Early Detection and Intervention Evaluation; MCQ‐30: Metacognitions Questionnaire‐30. Standard deviation in parentheses.

Respondents on these measures are required to select a number ranging from 1 *Do not agree* to 4 *Agree very much* for each of the items in the measure. A score is calculated for each of the five subscales as well as a total score for the whole measure. Internal consistency (as measured by Cronbach's alpha) for the original five‐factor structure in the current study was as follows: cognitive confidence α = 0.88, positive beliefs about worry α = 0.85, cognitive self‐consciousness α = 0.82, negative beliefs about uncontrollability and danger α = 0.83, negative beliefs about the need to control thoughts α = 0.75, and for the full measure α = 0.90. These results demonstrate that the internal consistency for the original five‐factors in an ARMS sample was acceptable to high.

The Comprehensive Assessment of At Risk Mental States (CAARMS; Yung et al., 2005) was administered in the EDIE‐2 trial to assess for at risk for psychosis status. Four of the six subscales in the CAARMS are used to determine ARMS status: unusual thought content (e.g., thought insertion, feeling controlled by something other than self); nonbizarre ideas (e.g., paranoid thoughts, feeling that one does not exist or is dead); perceptual abnormalities (e.g., visual, auditory, or sensory hallucinations); and disorganized speech (e.g., trouble finding the right word, tangential speech). In the version of the CAARMS used in EDIE‐2 (Yung et al., 2005), a Global Assessment of Functioning score was also calculated as problems with functioning was part of the criteria for ARMS. This version of the CAARMS was found to have very good validity and reliability (Yung et al., 2005). Further, interrater reliability checks were conducted eight times throughout the EDIE‐2 trial with good reliability found between raters (intraclass correlation coefficient = 0.90, *SD* = 0.03; Morrison et al., 2012).

The Positive and Negative Symptoms Scale (PANSS; Kay, Fiszbein, & Opler, 1987) was used in the EDIE trial to assess for at risk for psychosis status. Scores on the hallucinations, delusions, suspiciousness, and conceptual disorganisation subscales of the PANSS were used to determine if participants met criteria for an ARMS. This measure has been found to be reliable and valid (Kay et al., 1987).

The BAPS is an 18‐item self‐report assessment tool used in the EDIE‐2 trial to assess metacognitive beliefs about paranoia. The initial version of the BAPS had four subscales (Morrison et al., 2005). The revised three subscale version of this measure (Gumley et al., 2011) was used with participants included in this study. Internal consistency for the current sample for each subscale were negative beliefs about paranoia α = 0.88, survival beliefs about paranoia α = 0.87, and normalizing beliefs about paranoia α = 0.88.

The SIAS is a 20‐item self‐report questionnaire used to measure social anxiety (Mattick & Clarke, 1998). The SIAS was used for measurement of social anxiety in the EDIE‐2 trial. The Cronbach's alpha for the current sample was α = 0.90 demonstrating high reliability.

The BDI‐7 includes seven self‐report items designed to measure depression (Beck, Guth, Steer, & Ball, 1997) and was the depression measure used in EDIE‐2. Internal consistency for this study was very good: α = 0.86.

### 2.3 Data analyses

IBM SPSS AMOS Version 22 (AMOS) was used to run confirmatory factor analysis (CFA) to assess the goodness of fit of the original five‐factor structure of the MCQ‐30 and explore alternative solutions suggested by other analyses. A CFA was conducted first because the MCQ‐30 had an existing structure established in past research, and we aimed to test the hypothesis that the same five‐factor structure would be a good model fit for those with an ARMS (i.e., test construct validity).

It is recommended that several fit indices are used to assess model fit and should consist of the following: chi‐square and degrees of freedom (DF); an absolute fit index (e.g., goodness of fit index [GFI], standardized root mean residual [SRMR], and root mean square error of approximation [RMSEA]); one incremental fit index (e.g., Tucker–Lewis index [TLI] and comparative fit index [CFI]); a goodness of fit index (e.g., GFI, TLI, and CFI); and a badness of fit index (e.g., SRMR and RMSEA; Hair, Black, Babin, & Anderson, 2014). We used each of these indices to assess model fit allowing for a comprehensive analysis of fit and to reduce the risk of selection bias of fit indices that indicate a better fit.

Principal components analysis (PCA) was conducted in IBM SPSS Statistics Version 22 (SPSS) after completing the CFA. PCA is a factor analytic technique used to reduce data into meaningful groups or factors. In this study, it was used to explore potential alternative solutions of the MCQ‐30 and their factor loadings. Oblique rotation (direct oblimin) was used to allow for more flexibility in the position of factors (Kline, 1994) because past validation research on the MCQ‐30 (Wells & Cartwright‐Hatton, 2004) has demonstrated items on this measure correlate. Eigenvalues above 1 were retained for the initial exploration of the measure. We examined the scree plot (Cattell, 1966) to determine the number of factors to extract.

Parallel analysis is an alternative statistical method to determine the optimal number of factors to extract. It is recommended that this method is used in addition to the scree plot (O'Connor, 2000). Parallel analysis compares the eigenvalues of raw data to randomly selected data. Random data matches raw data in terms of the number of variables and observations (O'Connor, 2000). O'Connor (2000) suggests that random data are generated using the 95th percentile of the distribution of these randomly generated eigenvalues. Where the eigenvalue for the raw data is larger than the eigenvalue of the randomly generated data, the factor or component can be retained. A parallel analysis was run in SPSS using the O'Connor (2000) syntax; number of data sets input as 1,000; percentile set at 95; option “1” for PCA; and option “1” for normally distributed random data generation parallel analysis.

A further CFA was conducted in AMOS to test the model fit of an alternative factor structure identified by the parallel analysis to allow us to compare the results to the original five‐factor structure. Only participants that did not have any missing data in the MCQ‐30 (or MCQ‐30 items extracted from the MCQ) were included in factor analyses (i.e., no estimates were created for missing data).

SPSS was also used to calculate means and *SD*s of measures and to conduct one‐way MANOVA analyses to check if any statistical differences existed between males and females on the MCQ‐30 subscales and total scores.

Cronbach alphas are calculated in SPSS to measure internal consistency of measures (i.e., how correlated items are in a subscale to assess how much items measure the same construct).

SPSS was used to run Pearson correlations to test for convergent and discriminant validity. Only EDIE‐2 data (*N* = 153) was used for correlation analyses as the EDIE data did not have these measures. Correlations were generated using the pairwise option in SPSS to prevent complete exclusion of participants from correlation analyses where they had missing data from only some measures.

## 3 RESULTS

### 3.1 Sample

The mean age of the combined EDIE and EDIE‐2 sample (*N* = 185) was 20.54 years, minimum 14 years, maximum 34 years, and *SD* 4.06 years.

### 3.2 Confirmatory factor analysis

The chi‐square was found to be significant *X* ^*2*^ (395) = 683.45, *p* < 0.001, which indicates a poor fitting model. However, the chi‐square statistic is very sensitive to sample sizes (Garver & Mentzer, 1999; Hair, et al., 2014) and models with larger number of observed variables (individual items; Hair et al., 2014). The CMIN/DF (relative chi‐square/degrees of freedom) score for this analysis was 1.73, well below the threshold of <3 (Hoe, 2008). A score of <2 on this statistic indicates a very good fitting model (Hair et al., 2014). The RMSEA was also found to be within acceptable parameters at 0.06, as it was <0.08 (Garver & Mentzer, 1999). On the CFI, GFI, and TLI, a score of more than 0.90 indicates a good fitting model (Garver & Mentzer, 1999). Scores on these indicators were; CFI 0.87, TLI 0.86, and GFI 0.81. CFI and TLI scores were just below the cut‐off indicating a fair fitting model, whereas the GFI was further below the cut‐off. Finally, the SRMR score is recommended to be below 0.08 to indicate good fit (Hu & Bentler, 1999), and this was achieved with a value of 0.07.

In this model, most of the standardized regression weights were above the recommended cut‐off of 0.50 (Hair et al., 2014) with a range from 0.50 to 0.79. Only one item was found to be less than 0.50 from the cognitive self‐consciousness subscale (“I am aware of the way my mind works when I am thinking through a problem” = 0.44). Small to moderate correlations existed between most of the latent factors (ranges between 0.24 and 0.52). Larger correlations were found for the following subscales: need to control thoughts and cognitive self‐consciousness (0.66) and need to control thoughts and negative beliefs about uncontrollability and danger (0.68).

**Figure 1:**
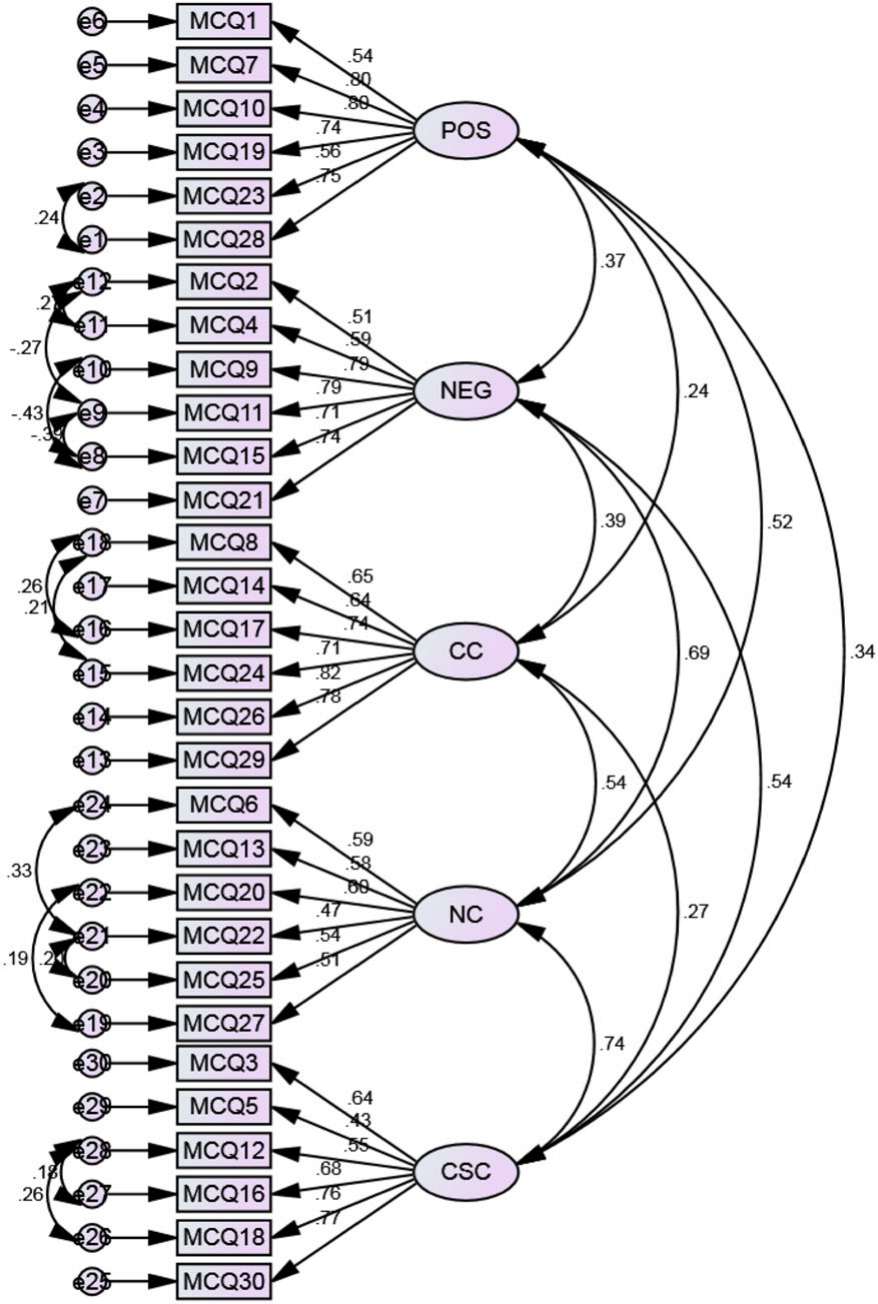
Final model for five‐factor confirmatory factor analysis with all errors correlated (standardized estimates). *N* = 185. Ovals represent Metacognitions Questionnaire‐30 (MCQ‐30) subscales (POS: positive beliefs about worry; NEG: negative beliefs about uncontrollability and danger; CC: cognitive confidence; NC: negative beliefs about the need to control thoughts; CSC: cognitive self‐consciousness). Boxes represent MCQ‐30 items (e.g., MCQ1: Question 1 of MCQ‐30 measure). Circles: errors. Double headed curved arrows: correlations. Straight arrows from subscales to items: regression weights [Colour figure can be viewed at http://wileyonlinelibrary.com]

Modification indices suggested the correlation of a number of errors within the same subscale. This improved the model as follows: *X* ^*2*^ (383) = 582.72, *p* < 0.001; CMIN/DF was 1.52; RMSEA = 0.05; CFI = 0.91; TLI = 0.90; GFI = 0.84; and SRMR = 0.07. Figure 1 shows the final model with correlated errors.

### 3.3 Principal component analysis

The Kaiser–Meyer–Olkin measure of sampling adequacy score for the sample was 0.85, which is considered to be very good. A score above 0.80 suggests that the sample size is sufficient for a PCA (Kaiser, 1974). Bartlett's test of sphericity *X* ^*2*^ = 2535.902 was very significant at the *p* < 0.0001 level, adding further support for the suitability of PCA for this sample.

Examination of the scree plot suggested a five‐factor model of the MCQ‐30 in the ARMS population. The PCA was rerun setting the fixed number of factors to five. Loadings on the pattern and structure matrices were checked. Where an item loaded on more than one factor and the difference between the loadings was equal to or more than 0.20, the highest loading item was retained on the factor it loaded highest on. Loadings at 0.40 or above were retained on each factor (Hair et al., 2014). Table 2 shows the structure matrix for all five factors.

**Table 2:** Principal component analysis structure matrix (five‐factor structure)

MCQ‐30 items	Factor loadings				
	1	2	3	4	5
Factor 1: Negative beliefs about uncontrollability and danger					
4. I could make myself sick with worrying	**0.784**	0.116	0.188	0.227	−0.134
21. When I start worrying, I cannot stop	**0.769**	0.211	0.191	0.186	−0.427
9. My worrying thoughts persist, no matter how I try to stop them	**0.714**	0.231	0.257	0.361	−0.409
2. My worrying is dangerous for me	**0.693**	0.160	0.049	0.256	−0.066
11. I cannot ignore my worrying thoughts	**0.672**	0.198	0.281	0.305	−0.441
15. My worrying could make me go mad	**0.635**	0.307	0.202	0.247	−0.357
Factor 2: Cognitive confidence					
17. I have a poor memory	0.107	**0.833**	0.071	0.054	−0.184
8. I have little confidence in my memory for words and names	0.077	**0.806**	0.105	0.143	−0.121
24. I have little confidence in my memory for places	0.229	**0.797**	0.161	0.090	−0.164
26. I do not trust my memory	0.171	**0.796**	0.132	0.149	−0.425
29. I have little confidence in my memory for actions	0.269	**0.791**	0.188	0.086	−0.275
14. My memory can mislead me at times	0.244	**0.647**	0.327	0.360	−0.348
Factor 3: Positive beliefs					
28. I need to worry in order to work well	0.100	0.189	**0.821**	0.157	−0.233
10. Worrying helps me to get things sorted out in my mind	0.124	0.081	**0.819**	0.231	−0.298
7. I need to worry in order to remain organized	0.293	0.132	**0.808**	0.270	−0.231
19. Worrying helps me cope	0.215	0.099	**0.777**	0.301	−0.174
23. Worrying helps me to solve problems	−0.023	0.223	**0.687**	0.112	−0.213
1. Worrying helps me to avoid problems in the future	0.261	0.124	**0.612**	0.250	−0.031
Factor 4: Cognitive self‐consciousness					
18. I pay close attention to the way my mind works	0.341	0.143	0.277	**0.825**	−0.174
16. I am constantly aware of my thinking	0.208	0.137	0.204	**0.776**	−0.265
30. I constantly examine my thoughts	0.338	0.245	0.094	**0.760**	−0.337
12. I monitor my thoughts	0.134	0.097	0.312	**0.720**	−0.125
3. I think a lot about my thoughts	0.504	0.164	0.131	**0.599**	−0.286
13. I should be in control of my thoughts all of the time	0.088	0.290	0.205	**0.579**	**−0.520** a
5. I am aware of the way my mind works when I am thinking through a problem	0.199	−0.089	0.212	**0.520**	−0.181
Factor 5: Negative beliefs about the need to control thoughts					
22. I will be punished for not controlling certain thoughts	0.281	0.259	0.211	0.154	**−0.741**
6. If I did not control a worrying thought, and then it happened, it would be my fault	0.404	0.202	0.304	0.286	**−0.688**
20. Not being able to control my thoughts is a sign of weakness	0.323	0.318	0.102	0.369	**−0.647**
25. It is bad to think certain thoughts	0.333	0.166	0.376	0.299	**−0.560**
27. If I could not control my thoughts, I would not be able to function	0.123	0.251	0.255	**0.416**	**−0.534**

*Note*. MCQ‐30: Metacognitions Questionnaire‐30. Bold = loadings >0.40. Underscore = higher scoring loading where a loading >0.40 loads on more than one factor.

a Loadings that score lower on their original subscale than another factor.

Factors 1, 2, and 3 of the analysis included all the items that matched exactly the original MCQ‐30 subscales: negative beliefs about uncontrollability and danger, cognitive confidence, and positive beliefs about worry, respectively. Where double loadings (i.e., items loading on another factor) were present on these three factors, the differences between the loadings was ≥0.20, and the higher loading was always on the original subscale. In this circumstance, the higher scoring item was retained and the lower scoring item ignored.

Factors 4 and 5 are related to subscales cognitive self‐consciousness and negative beliefs about the need to control thoughts, respectively. All items related to the original subscale of the MCQ‐30 loaded on these two factors. However, three variables (Questions 3, 13, and 27) loaded on two factors of the MCQ‐30. The differences between the two loadings in each case was <0.20. Loadings for Questions 3 and 27 were slightly higher for the original subscale, and for Question 13, it was slightly higher for the factor that did not relate to the original subscale.

Further investigation into the content of these questions and where they were loading suggested that the double loadings were logical and made theoretic sense. Question 13 (“I should be in control of my thoughts all of the time”), for example, loaded slightly higher on Factor 4 (cognitive self‐consciousness) rather than its original subscale negative beliefs about the need to control thoughts (Factor 5). This double loading is consistent with the high correlation between the need to control thoughts and cognitive self‐consciousness latent factors in the CFA. It makes sense that if someone believes they should be in control of their thoughts, they are likely to constantly monitor them so that this may be achieved.

### 3.4 Parallel analysis

The parallel analysis output suggested a four‐factor solution. Due to this, a PCA was rerun in SPSS using oblique rotation, setting the fixed number of factors to four. Factors 2 and 3 included items that matched exactly the original subscales for cognitive confidence and positive beliefs about worry, respectively. No additional items were included on these two factors. Factor 1 included all items in the original MCQ‐30 for the negative beliefs about uncontrollability and danger of worry subscale. Factor 4 included all items for the cognitive self‐consciousness subscale. However, one of the items under Factor 4 (Question 3: “I think a lot about my thoughts”) also loaded on Factor 1. This loaded higher on Factor 4 (its original subscale), but the difference between the items was <0.20. Again this loading appeared logical because if a belief exists that thoughts are uncontrollable and dangerous, then an increase in the amount of time thinking about thoughts is likely.

The subscale need to control thoughts did not emerge as a factor in its own right. Instead, the items for this subscale were split between Factors 1 (negative beliefs about uncontrollability and danger) and 4 (cognitive self‐consciousness). Factor 1 had four items loaded (Questions 6, 20, 22, and 25) and Factor 4 had two items loaded (Questions 13 and 27). The loading of items onto these two factors is reflected in the high correlations (0.69 and 0.74, respectively) generated in the CFA model (Figure 1) between these subscales. Overall, the PCA showed very few cross loadings existed. Where cross loadings were present, they were minor and seemed theoretically coherent.

Although the parallel analysis suggests a four‐factor structure, examination of the loading of the items did not suggest an alternative structure that was theoretically coherent. However, as a final test, a further CFA was run to examine the construct validity of this four‐factor structure. The chi‐square was found to be significant *X* ^*2*^ (399) = 740.16, *p* < 0.001; CMIN/DF was 1.86; RMSEA = 0.07; CFI = 0.85; TLI = 0.84; GFI = 0.79; and SRMR = 0.08. Modification indices improved the model as follows: *X* ^*2*^ (385) = 606.83, *p* < 0.001; CMIN/DF was 1.58; RMSEA = 0.06; CFI = 0.90; TLI = 0.89; GFI = 0.83; and SRMR = 0.07. However, the results illustrate the four‐factor structure had a poorer model fit than the original five‐factor structure.

### 3.5 MCQ‐30 descriptive statistics for original five‐factor structure

Means and *SD*s were calculated for the five original MCQ‐30 subscales. Table 3 shows the results of this analysis for the total sample and split across males and females. A one‐way MANOVA found no significant differences between males and females on any of the five MCQ‐30 subscales or the MCQ‐30 total score.

**Table 3:** MCQ‐30 means and *SD*s for combined EDIE and EDIE‐2 data

MCQ‐30 subscale	Mean total sample	Mean male	Mean female
	(*N* = 185)	(*n* = 112)	(*n* = 73)
Cognitive confidence	12.59 (4.96)	12.46 (4.96)	12.79 (4.98)
Positive beliefs about worry	10.43 (4.01)	10.55 (3.82)	10.23 (4.30)
Cognitive self‐consciousness	15.69 (4.35)	15.79 (4.39)	15.53 (4.31)
Negative beliefs about uncontrollability and danger	15.03 (4.83)	14.44 (4.71)	15.93 (4.92)
Negative beliefs about the need to control thoughts	12.97 (4.24)	12.74 (4.01)	13.33 (4.58)
MCQ‐30 Total score	66.71 (15.75)	65.99 (14.78)	67.82 (17.17)

*Note*. EDIE: Early Detection and Intervention Evaluation; MCQ‐30: Metacognitions Questionnaire‐30; *SD*: standard deviation. *SD* in parentheses.

The highest scoring subscale for the total sample was cognitive self‐consciousness followed by negative beliefs about uncontrollability and danger. This is in line with past ARMS research using the MCQ‐30 (Palmier‐Claus et al., 2013). Females, however, scored highest on negative beliefs about uncontrollability and danger followed by cognitive self‐consciousness. Past ARMS research found negative beliefs about uncontrollability and danger to be the highest scoring subscale followed by cognitive self‐consciousness in the total sample (Welsh et al., 2014).

### 3.6 Internal consistency

Cronbach's alphas for the MCQ‐30 in this sample (reported in the measures section) ranged from acceptable to high in this ARMS sample. Separate Cronbach's alphas were also calculated for males (m: *n* = 112) and females (f: *n* = 73) for all subscales and the full MCQ‐30: positive beliefs about worry α = 0.82 (m) and 0.89 (f), negative beliefs about uncontrollability and danger α = 0.82 (m) and 0.84 (f), cognitive confidence α = 0.88 (m) and 0.88 (f), need to control thoughts α = 0.70 (m) and 0.81 (f), cognitive self‐consciousness α = 0.82 (m) and 0.81 (f), and for the full MCQ‐30 α = 0.89 (m) and 0.92 (f). Most of the scores represented good to excellent internal consistency, and in all but one subscale (need to control thoughts) males and females had similar scores. The male score for need to control thoughts was at 0.70, which was an acceptable level. The female score had a higher internal consistency (0.81).

### 3.7 Convergent and discriminant validity

Convergent validity of the MCQ‐30 was tested by correlating the subscales and total score with related concepts: the BAPS, BDI‐7, and SIAS. The mean age for this EDIE‐2 subsample was 20.17 years, minimum 14 years, maximum 34 years, *SD* 3.96 years, and the male to female ratio 88:65. The ethnicity distribution for this sample was as follows: White: *n* = 136 (88.9%), Black Caribbean: *n* = 1 (0.7%), Black African: *n* = 4 (2.6%), Indian: *n* = 1 (0.7%), Pakistani: *n* = 3 (2%), Chinese: *n* = 1 (0.7%), other: *n* = 3 (2%), and not known: *n* = 4 (2.6%). Descriptives for measures are shown in Table 4. The correlation results are shown in Table 5.

**Table 4:** Means and *SD*s for EDIE‐2 measures

Measure	Mean	Minimum	Maximum
BDI‐7 Total	7.48 (5.01)	0	19
SIAS Total	37.49 (17.26)	5	73
BAPS Negative beliefs	14.37 (5.70)	0	24
BAPS Survival strategy	10.15 (4.67)	0	24
BAPS Normalizing beliefs	15.20 (5.71)	0	24

*Note*. BAPS: Beliefs about Paranoia Scale; BDI‐7: Beck Depression Inventory‐7; EDIE: Early Detection and Intervention Evaluation; SIAS: Social Interaction Anxiety Scale; *SD*: standard deviation. *SD* in parentheses.

**Table 5:** Correlation matrix for EDIE‐2 data

	2	3	4	5	6	7	8	9	10	11
1. Cognitive confidence	0.26**	0.21**	0.32**	0.46**	0.65**	0.20*	0.15	0.01	0.40**	0.40**
2. Positive beliefs about worry	—	0.31**	0.28**	0.42**	0.62**	0.26**	0.38**	0.13	0.19*	0.35**
3. Cognitive self‐consciousness		—	0.46**	0.55**	0.70**	0.30**	0.27**	0.11	0.34**	0.30**
4. Negative beliefs about uncontrollability and danger			—	0.55**	0.75**	0.53**	0.05	−0.04	0.56**	0.38**
5. Need to control thoughts				—	0.83**	0.44**	0.21*	0.13	0.50**	0.35**
6. MCQ‐30 Total score					—	0.49**	0.29**	0.09	0.56**	0.51**
7. BAPS Negative beliefs						—	0.38**	0.27**	0.46**	0.51**
8. BAPS Survival strategy							—	0.39**	0.26**	0.38**
9. BAPS Normalizing beliefs								—	−0.03	−0.01
10. BDI‐7 Total									—	0.55**
11. SIAS Total										—

*Note*. BAPS: Beliefs about Paranoia Scale; BDI‐7: Beck Depression Inventory‐7; EDIE: Early Detection and Intervention Evaluation; SIAS: Social Interaction Anxiety Scale; MCQ‐30: Metacognitions Questionnaire‐30.

* 0.05 level.

** 0.01 level.

A significant large positive relationship was found between negative beliefs about uncontrollability and danger of worry subscale of the MCQ‐30 and the BAPS negative subscale (*N* = 153). Further, a moderate to large positive relationship was found between the negative beliefs about the need to control thoughts MCQ‐30 subscale and the BAPS negative subscale (*N* = 153). A moderate correlation was found between the positive beliefs about worry subscale and the BAPS survival subscale (*N* = 153). Significant large positive effects were found between the total MCQ‐30 scores and the SIAS (*N* = 142) and BDI‐7 (*N* = 152).

Discriminant validity was tested by correlating the MCQ‐30 with the BAPS normalizing beliefs subscales. No significant relationships were found between any of the MCQ‐30 subscales and this measure as predicted.

## 4 DISCUSSION

With the increased use of the MCQ‐30 measure in at risk for psychosis research, it is important to examine the validity of this measure in this sample. The fit and structure of the original five‐factor model was tested using CFA. Although the chi‐square result indicated a poor fitting model, the chi‐square is very sensitive to sample size (Garver & Mentzer, 1999; Hair et al., 2014), with larger samples leading to the increased likelihood of a significant chi‐square. Further, the chances of the chi‐square being significant is increased the more observed variables (i.e., individual items that make up each subscale or overall measure) there are in a model (Hair et al., 2014). It is, therefore, recommended that a range of fit indices are interpreted. The CMIN/DF (modified chi‐square) was used in addition to assess model fit and suggested a very good fit as it was well below the cut‐off. Further, the RMSEA was used in this study because it corrects for both sample size and complexity of model issues of the chi‐square and better shows how well a model fits a sample (Hair et al., 2014). The RMSEA fit index also demonstrated a good model fit for this sample. The TLI is not as sensitive to changes in sample size, and the CFI is less sensitive to the complexity of models (Hair et al., 2014). Initially, both these indices were just below the cut‐off indicating a fair fitting model. However, after correlating the errors both were within the cut‐off indicating a good model. Overall, the majority of the CFA output indicated a good fit of the original five‐factor structure of the MCQ‐30 in this ARMS sample using the most commonly used and recommended indices (Hair et al., 2014; Hoe, 2008; Hu & Bentler, 1999).

To explore other possible latent structures for the MCQ‐30, a PCA was run with a five‐factor solution suggested by the scree plot, which was almost identical to the original solution. However, a four‐factor solution was suggested by the results of parallel analysis. A four‐factor solution was, therefore, specified and the PCA rerun. All items loaded under their original subscales except the negative beliefs about the need to control thoughts subscale. The items for this measure were split between Factor 1, negative beliefs about uncontrollability and danger of thoughts (four items) and Factor 4, cognitive self‐consciousness (two items). However, these loadings made theoretical sense (e.g., if someone is feeling the need to control thoughts, they will likely increase monitoring of their thoughts hence the loading of negative items on the cognitive self‐consciousness subscale). Further, the CFA results established that the original five‐factor model had a better fit than the four‐factor model recommended by the parallel analysis. All fit indices for the five‐factor model showed a better fit including the SRMR, which is the statistic that is recommended for comparing models (Hair et al., 2014).

Convergent validity was tested by correlating the MCQ‐30 with depression, social anxiety, and beliefs about paranoia. Consistent with our hypotheses, significant positive relationships were found between the MCQ‐30 and depression and social anxiety, which fit with the findings of past research. Moderate to large positive relationships between the negative subscales of the MCQ‐30 and the negative beliefs subscale of the BAPS were also found. Positive beliefs about worry subscale of the MCQ‐30 and the survival subscale of the BAPS were significantly positively correlated. This would be expected as these subscales both concern beliefs about the usefulness of such worrying and paranoid thoughts (respectively). In line with our hypothesis, no significant correlation existed between the MCQ‐30 and the BAPS normalizing beliefs subscale. Negative beliefs about uncontrollability and danger and cognitive self‐consciousness subscales were the two highest scoring subscales of the MCQ‐30, a finding that fits with past ARMS research.

Despite the good fit illustrated by the comprehensive examination of various tests of validity and reliability, some limitations exist for this study. The use of secondary data meant that other tests of validity and reliability could not be run. Test–retest reliability, for example, was not examined because the study involved a psychological intervention that lasted for 6 months. This could have led to different responses at the 6‐month time point from participants who received treatment. With regards to criterion validity, which is described as how much scores on a measure relate to the gold standard (Terwee et al., 2007), as the MCQ‐30 is considered to be the gold standard measure of metacognitive beliefs, this test could not be carried out. Although measures that assess some related concepts such as the Thought Control Questionnaire (Wells & Davies, 1994) could have been utilized instead, the use of secondary data meant that this could not be tested in the current study.

Although the internal consistency scores for all MCQ‐30 subscales were between acceptable and very good, and males and females had similar Cronbach's alpha scores on four out of five of the subscales, there was a noticeable difference on the negative beliefs about the need to control thoughts subscale. Females scored higher on this subscale than males. It is not possible to determine the reasons for this from this study; however, past research has found that females tend to ruminate more than males (Bahrami & Yousefi, 2011; Johnson & Whisman, 2013). Due to this, it is possible that females may feel the need to control their thoughts more than males. The male internal consistency score was still within acceptable parameters, so it seems the difference does not have an effect on the overall reliability of the subscale in the context of this study. However, it would be worth exploring this difference in future studies to better understand the variance.

In summary, psychometric analysis appears to confirm that the original five‐factor structure of the MCQ‐30 is valid for measuring metacognitive beliefs in those with an ARMS. The MCQ‐30 with ARMS samples can be interpreted in the same way as in other psychological disorders. Future studies might find it useful to validate the MCQ‐30 in other samples across the psychosis spectrum (e.g., first episode psychosis) and consider testing theoretical models using this tool. Further tests of reliability and validity that could not be conducted in the current study should also be examined in future work. It might also be useful for CFA analyses to be conducted in larger samples to better establish the latent structure of the instrument and for closer examination of any gender differences on individual subscales of the measure.

## CONFLICT OF INTEREST

None.
